# Supplementary figures and images for: Icariin activates far upstream element binding protein 1 to regulate hypoxia-inducible factor-1α and hypoxia-inducible factor-2α signaling and benefits chondrocytes
Source: PeerJ. 2023 Aug 22;11:e15917. doi: 10.7717/peerj.15917 (PMC10452614; doi:10.7717/peerj.15917)

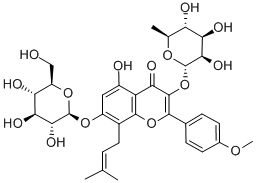

Supplement: Supplemental Information 1 [file peerj-11-15917-s001.zip › Raw data/figure 1/a/ICA.tif]

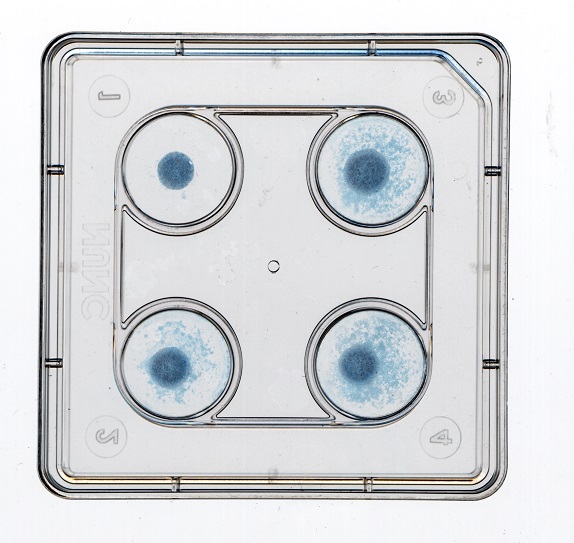

Supplement: Supplemental Information 1 [file peerj-11-15917-s001.zip › Raw data/figure 1/c/Alcian blue scan.jpg]

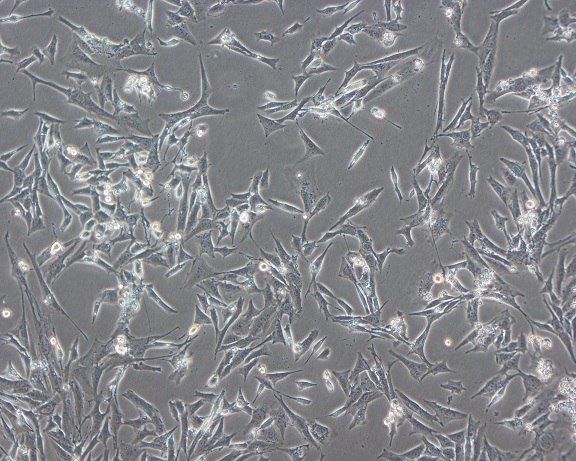

Supplement: Supplemental Information 1 [file peerj-11-15917-s001.zip › Raw data/figure 1/d/0.5 uM ICA-.jpg]

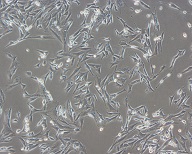

Supplement: Supplemental Information 1 [file peerj-11-15917-s001.zip › Raw data/figure 1/d/1 uM ICA-.jpg]

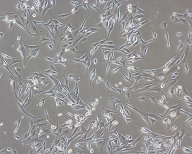

Supplement: Supplemental Information 1 [file peerj-11-15917-s001.zip › Raw data/figure 1/d/2 uM ICA-.jpg]

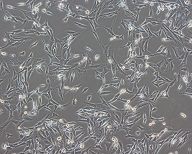

Supplement: Supplemental Information 1 [file peerj-11-15917-s001.zip › Raw data/figure 1/d/Con-.jpg]

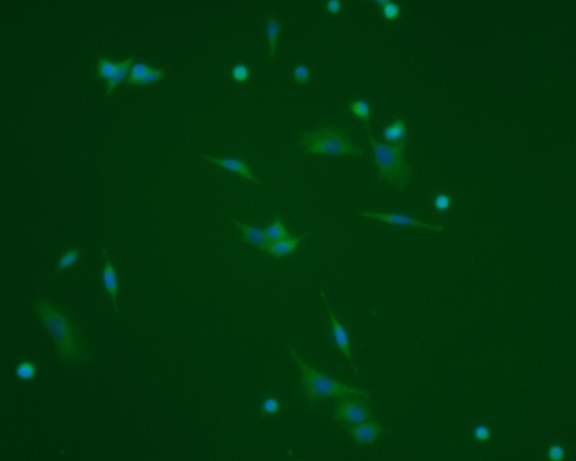

Supplement: Supplemental Information 1 [file peerj-11-15917-s001.zip › Raw data/figure 2/a/CON-Merged.jpg]

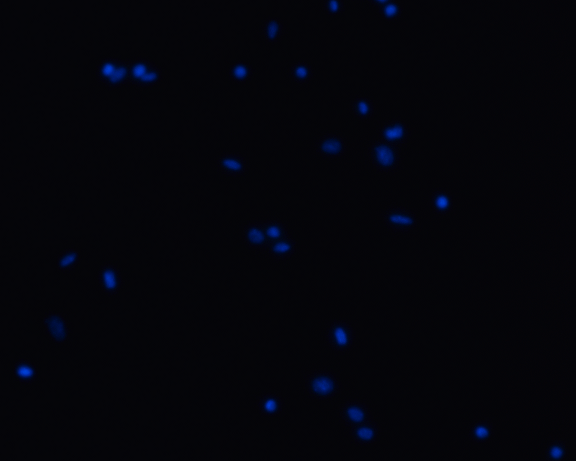

Supplement: Supplemental Information 1 [file peerj-11-15917-s001.zip › Raw data/figure 2/a/Con-DAPI.tif]

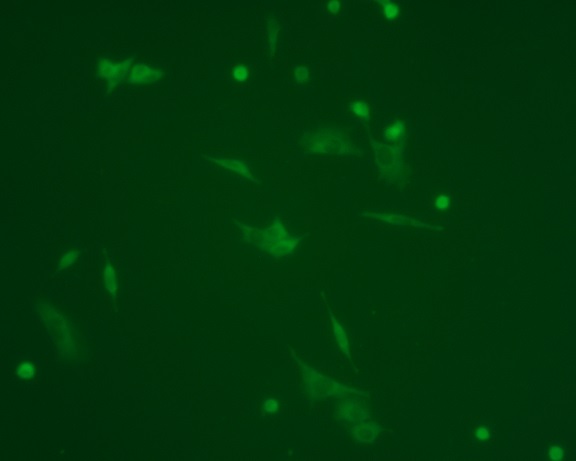

Supplement: Supplemental Information 1 [file peerj-11-15917-s001.zip › Raw data/figure 2/a/Con-FUBP1.jpg]

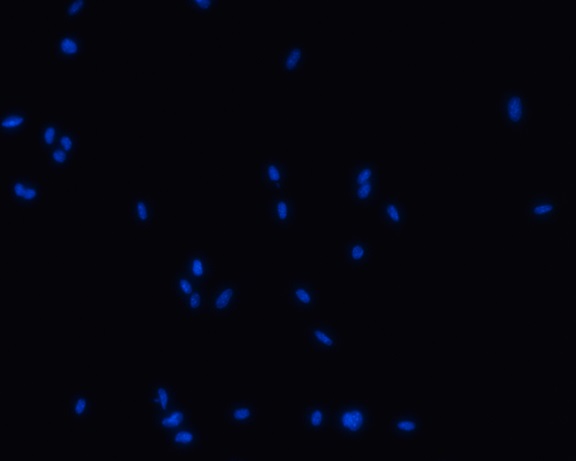

Supplement: Supplemental Information 1 [file peerj-11-15917-s001.zip › Raw data/figure 2/a/KD-DAPI.jpg]

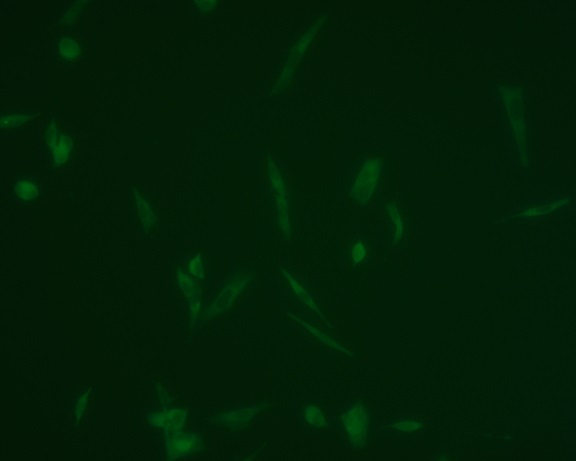

Supplement: Supplemental Information 1 [file peerj-11-15917-s001.zip › Raw data/figure 2/a/KD-FUBP1.jpg]

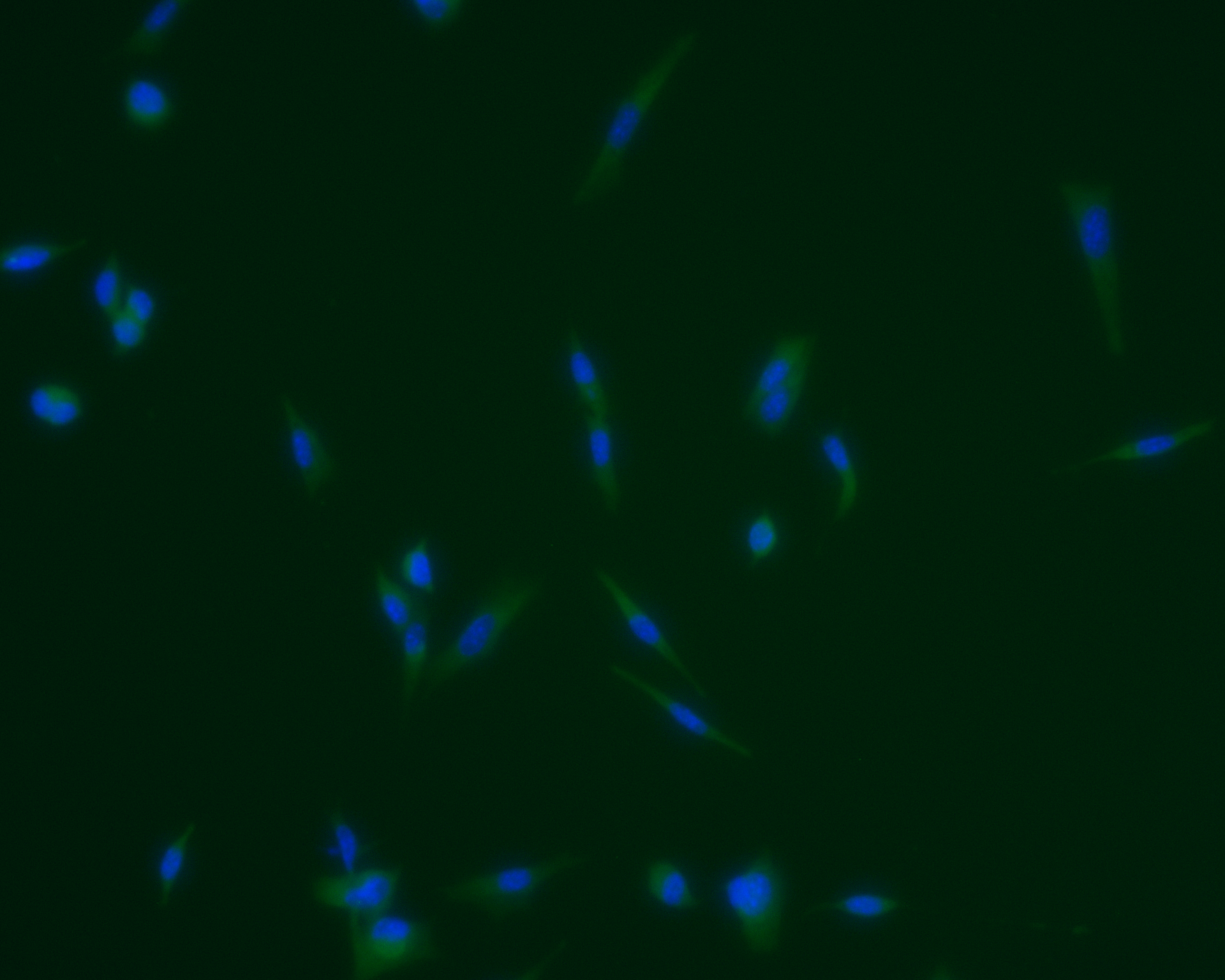

Supplement: Supplemental Information 1 [file peerj-11-15917-s001.zip › Raw data/figure 2/a/KD-Merge.tif]

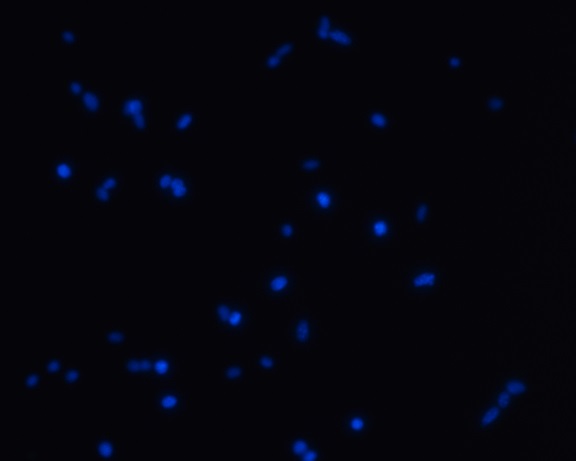

Supplement: Supplemental Information 1 [file peerj-11-15917-s001.zip › Raw data/figure 2/a/OE-DAPI.jpg]

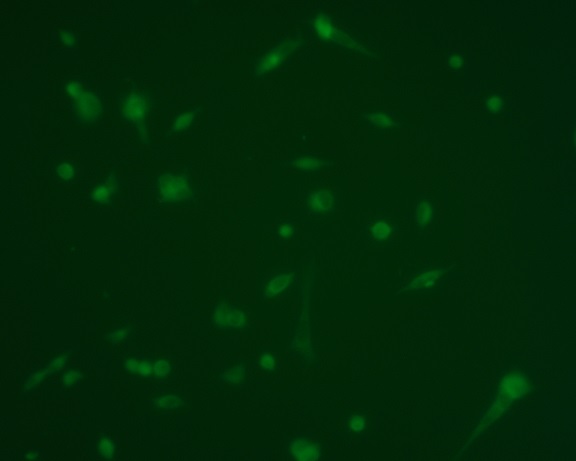

Supplement: Supplemental Information 1 [file peerj-11-15917-s001.zip › Raw data/figure 2/a/OE-FUBP1.jpg]

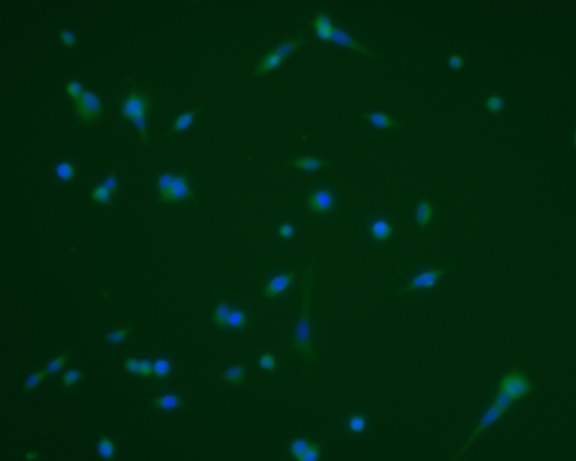

Supplement: Supplemental Information 1 [file peerj-11-15917-s001.zip › Raw data/figure 2/a/OE-Merge.jpg]

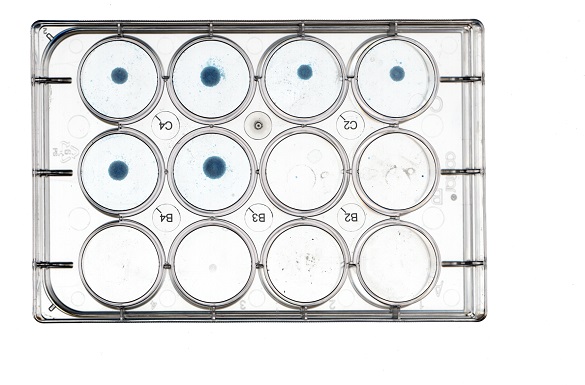

Supplement: Supplemental Information 1 [file peerj-11-15917-s001.zip › Raw data/figure 2/c/Alcian blue scan.jpg]

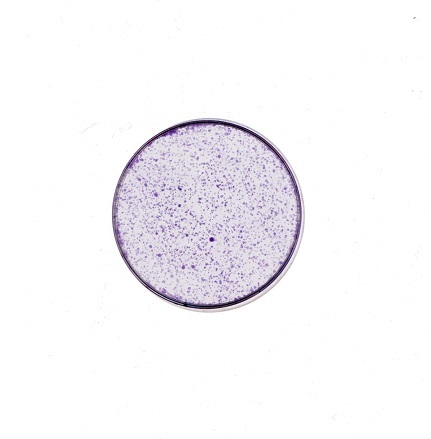

Supplement: Supplemental Information 1 [file peerj-11-15917-s001.zip › Raw data/figure 2/d/Con1.jpg]

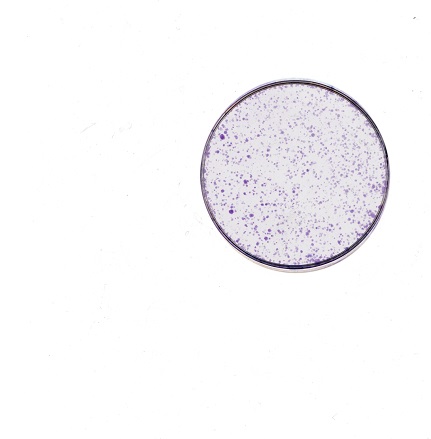

Supplement: Supplemental Information 1 [file peerj-11-15917-s001.zip › Raw data/figure 2/d/FUBP1-KD.jpg]

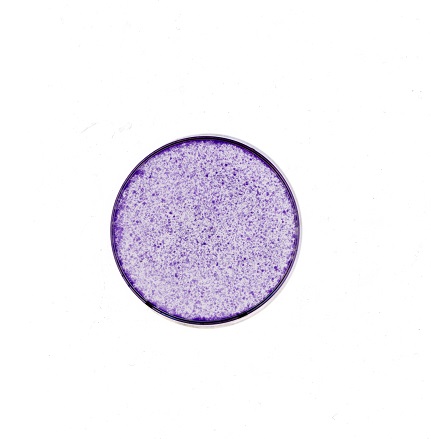

Supplement: Supplemental Information 1 [file peerj-11-15917-s001.zip › Raw data/figure 2/d/FUBP1-OE.jpg]

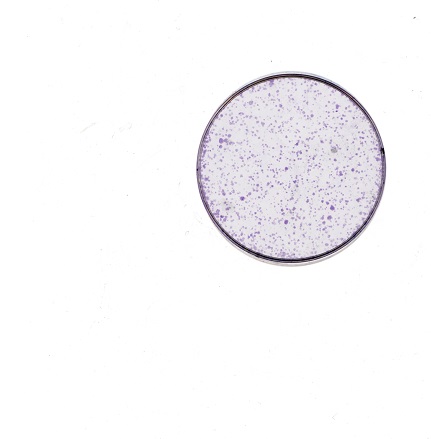

Supplement: Supplemental Information 1 [file peerj-11-15917-s001.zip › Raw data/figure 2/d/ICA-FUBP1-KD.jpg]

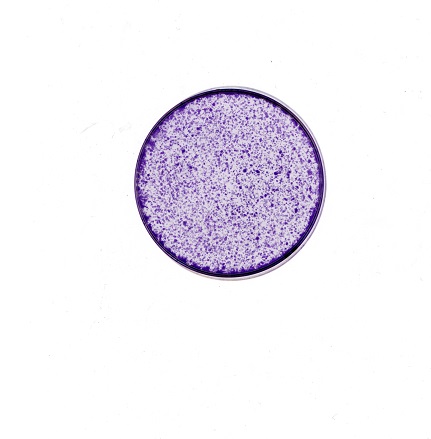

Supplement: Supplemental Information 1 [file peerj-11-15917-s001.zip › Raw data/figure 2/d/ICA-FUBP1-OE.jpg]

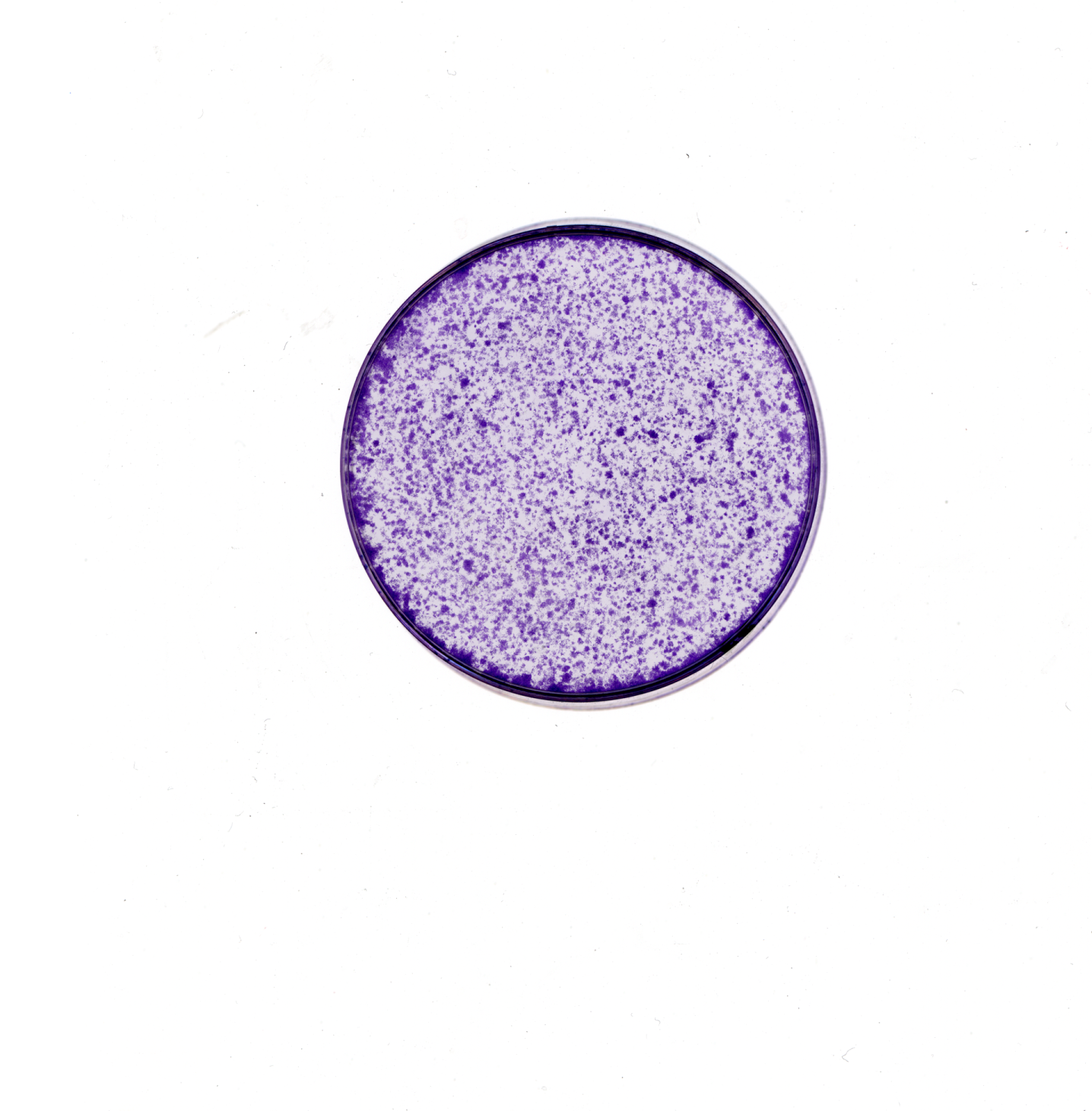

Supplement: Supplemental Information 1 [file peerj-11-15917-s001.zip › Raw data/figure 2/d/ICA-FUBP1-OE.tif]

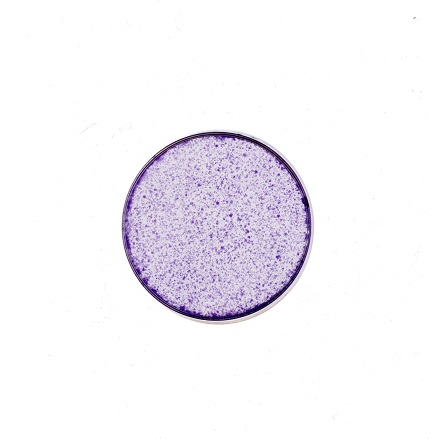

Supplement: Supplemental Information 1 [file peerj-11-15917-s001.zip › Raw data/figure 2/d/ICA.jpg]

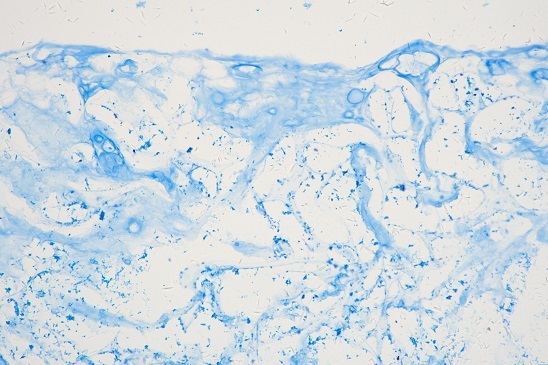

Supplement: Supplemental Information 1 [file peerj-11-15917-s001.zip › Raw data/figure 3/Alcian blue-Con.jpg]

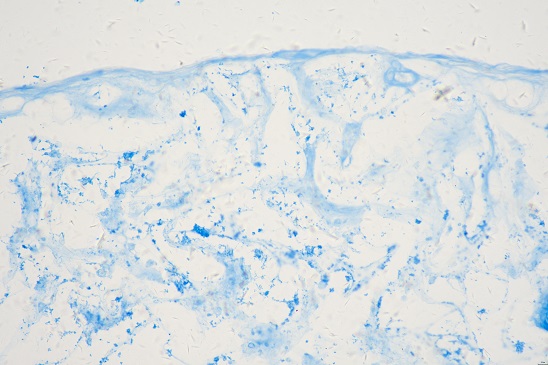

Supplement: Supplemental Information 1 [file peerj-11-15917-s001.zip › Raw data/figure 3/Alcian blue-FUBP1-KD.jpg]

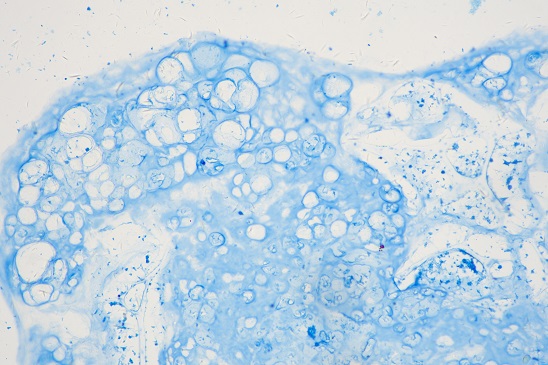

Supplement: Supplemental Information 1 [file peerj-11-15917-s001.zip › Raw data/figure 3/Alcian blue-FUBP1-OE.jpg]

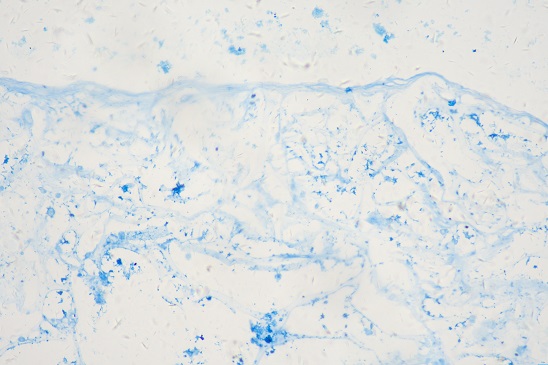

Supplement: Supplemental Information 1 [file peerj-11-15917-s001.zip › Raw data/figure 3/Alcian blue-ICA-FUBP1-KD.jpg]

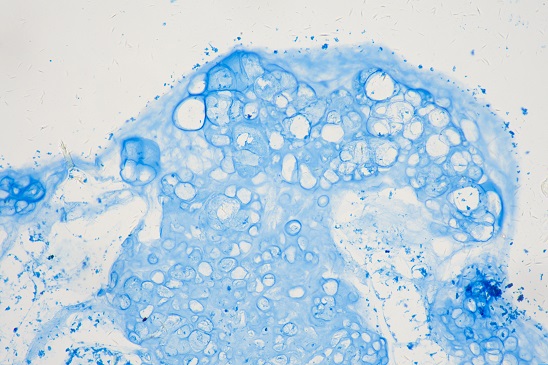

Supplement: Supplemental Information 1 [file peerj-11-15917-s001.zip › Raw data/figure 3/Alcian blue-ICA-FUBP1-OE.jpg]

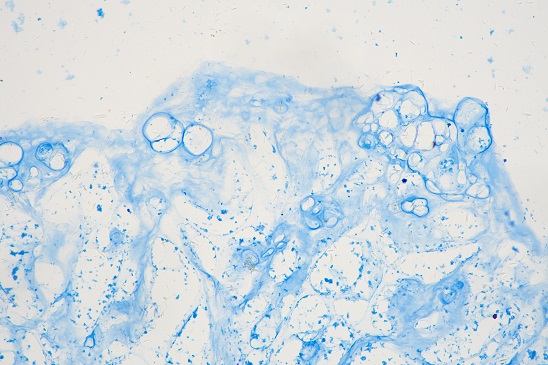

Supplement: Supplemental Information 1 [file peerj-11-15917-s001.zip › Raw data/figure 3/Alcian blue-ICA.jpg]

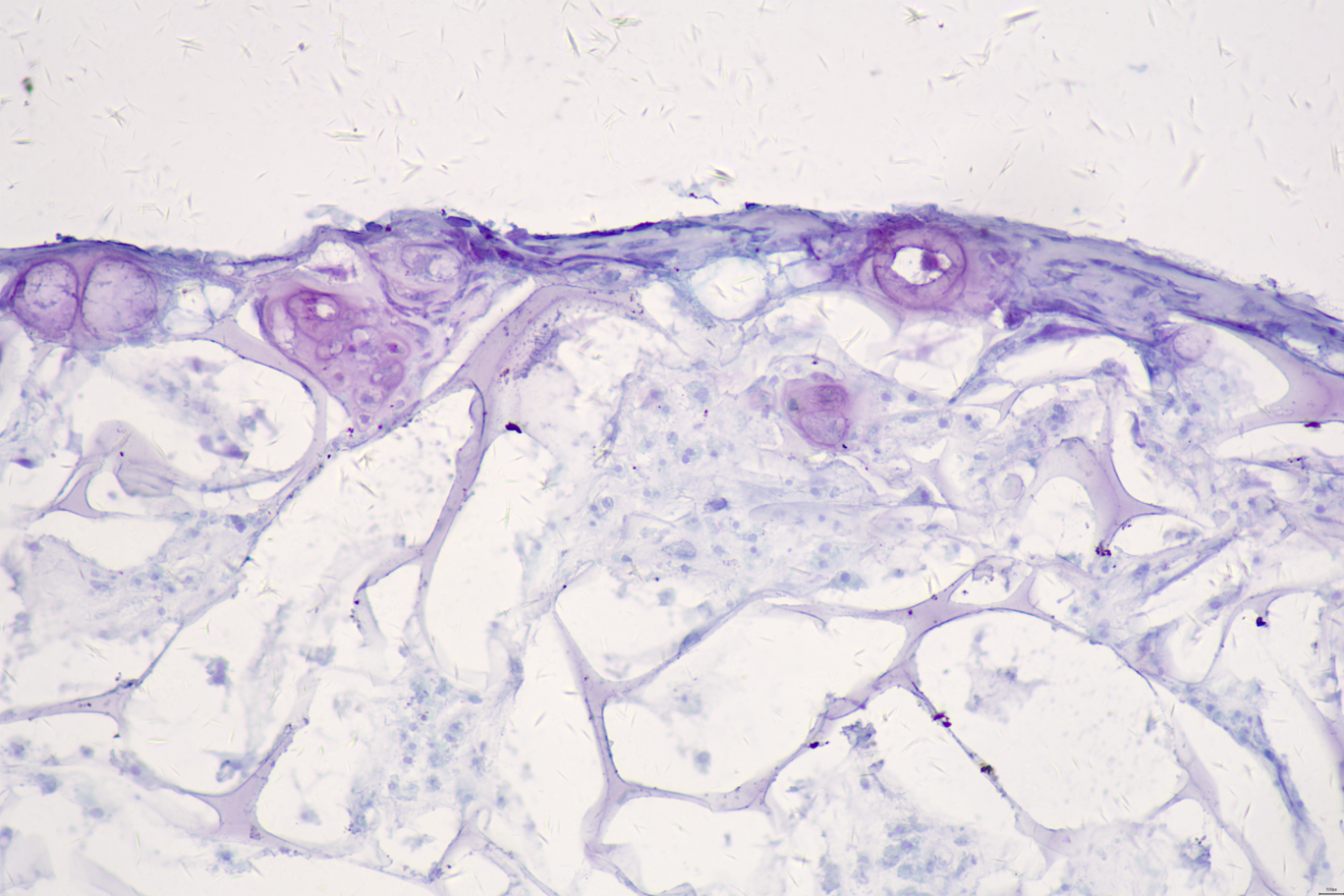

Supplement: Supplemental Information 1 [file peerj-11-15917-s001.zip › Raw data/figure 3/SO-Con.tif]

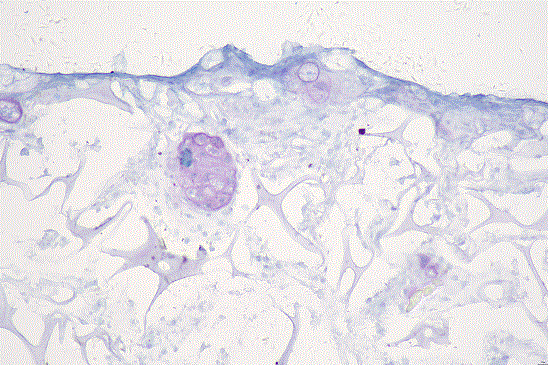

Supplement: Supplemental Information 1 [file peerj-11-15917-s001.zip › Raw data/figure 3/SO-FUBP1-KD.gif]

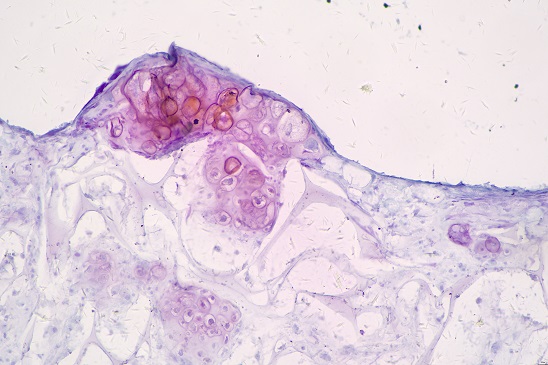

Supplement: Supplemental Information 1 [file peerj-11-15917-s001.zip › Raw data/figure 3/SO-FUBP1-OE.jpg]

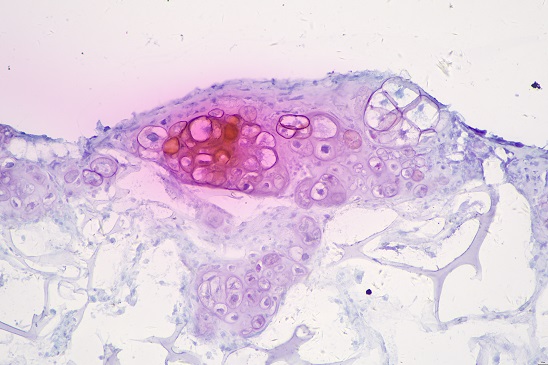

Supplement: Supplemental Information 1 [file peerj-11-15917-s001.zip › Raw data/figure 3/SO-ICA-FUBP1-OE.jpg]

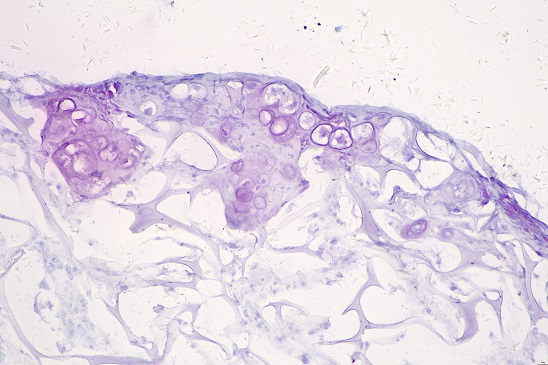

Supplement: Supplemental Information 1 [file peerj-11-15917-s001.zip › Raw data/figure 3/SO-ICA.jpg]

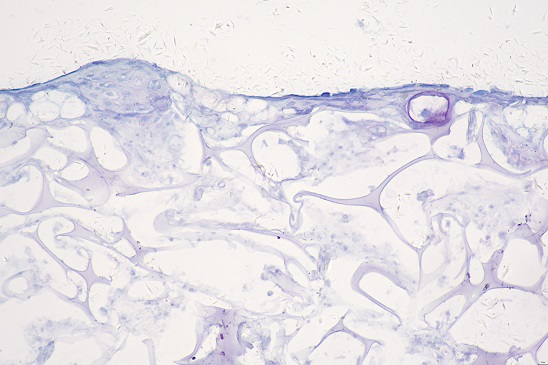

Supplement: Supplemental Information 1 [file peerj-11-15917-s001.zip › Raw data/figure 3/SO-ICAFUBP1-KD.jpg]

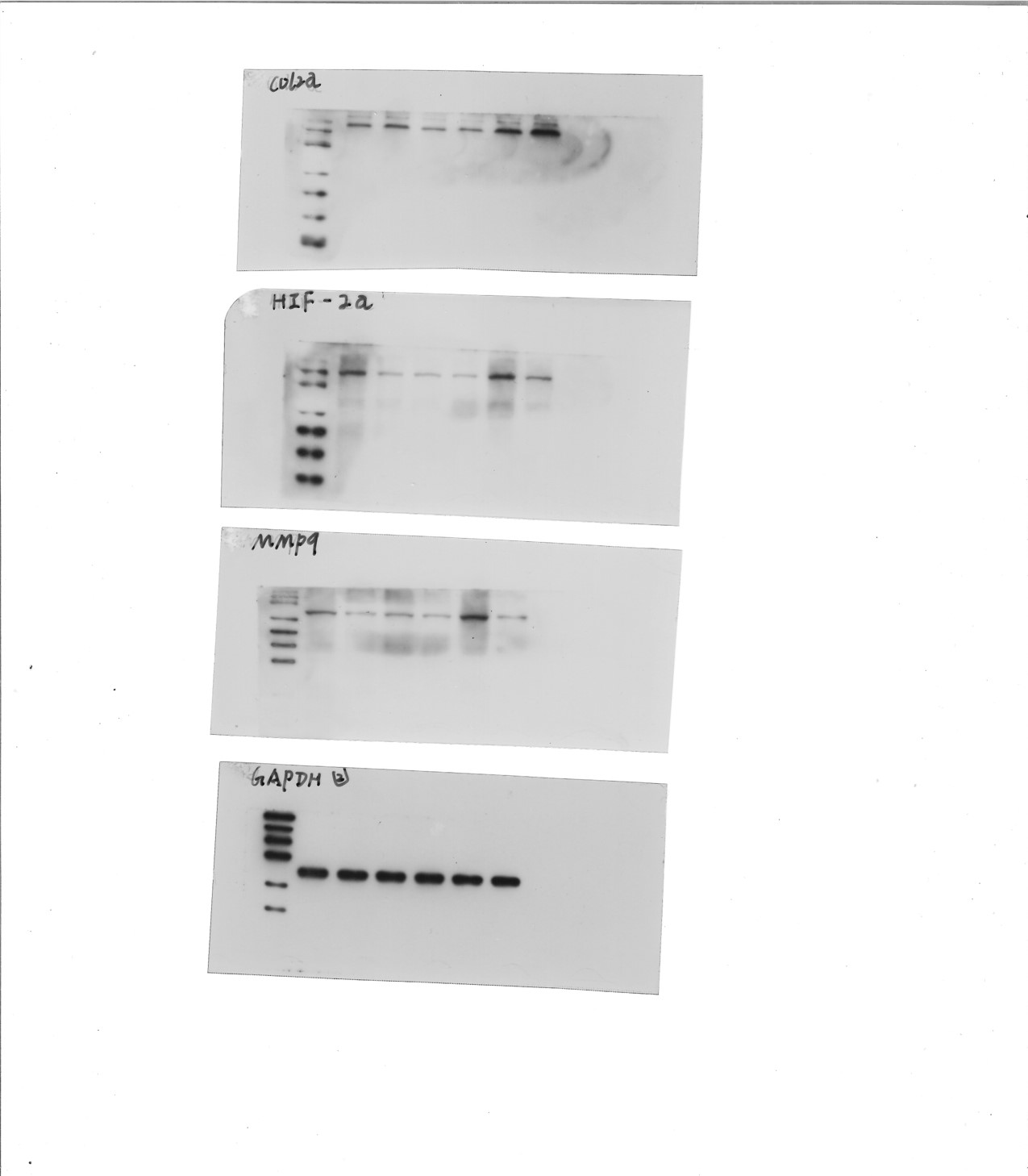

Supplement: Supplemental Information 1 [file peerj-11-15917-s001.zip › Raw data/figure 4/WB bands 2.jpg]

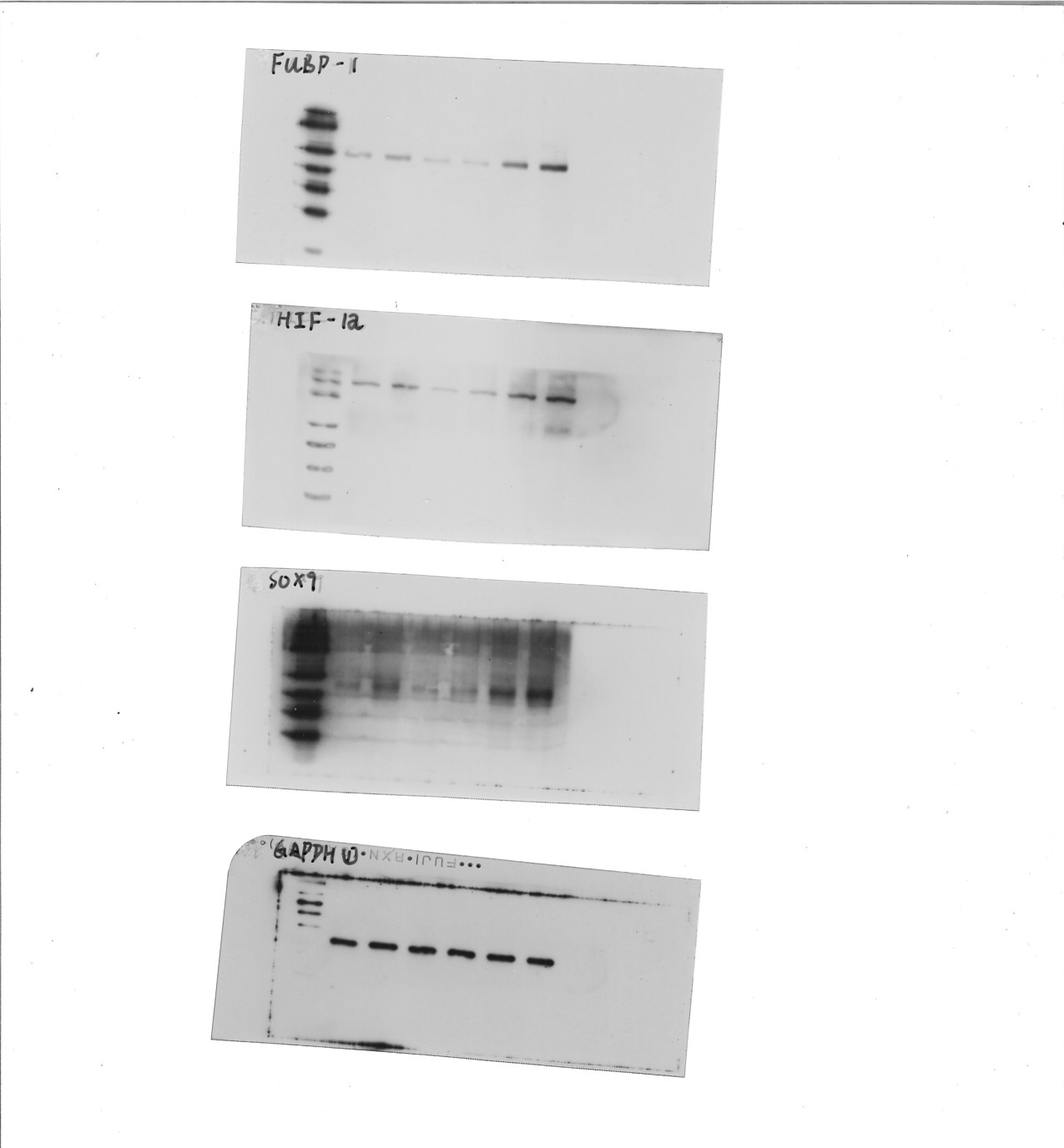

Supplement: Supplemental Information 1 [file peerj-11-15917-s001.zip › Raw data/figure 4/WB bans 1.jpg]

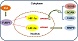

Supplement: Supplemental Information 1 [file peerj-11-15917-s001.zip › Raw data/figure 5/Figure 5.jpg]
